# Historical evolution of cancer genomics research in Latin America: a comprehensive visual and bibliometric analysis until 2023

**DOI:** 10.3389/fgene.2024.1327243

**Published:** 2024-01-18

**Authors:** Ivan David Lozada-Martinez, Luz Miryam Lozada-Martinez, Andrés Cabarcas-Martinez, Franklin Kevin Ruiz-Gutierrez, Jose Gabriel Aristizabal Vanegas, Katherine Julieth Amorocho Lozada, Lina María López-Álvarez, Ornella Fiorillo Moreno, Elkin Navarro Quiroz

**Affiliations:** ^1^ Epidemiology Program, Department of Graduate Studies in Health Sciences, Universidad Autónoma de Bucaramanga, Bucaramanga, Colombia; ^2^ Microbiology Program, Universidad Libre, Pereira, Colombia; ^3^ School of Medicine, Universidad de Cartagena, Cartagena, Colombia; ^4^ Department of Intensive Care, Hospital Felix Bulnes Cerda, Santiago, Chile; ^5^ School of Medicine, Universidad Industrial de Santander, Bucaramanga, Colombia; ^6^ Clinica Iberoamérica, Barranquilla, Colombia; ^7^ Clinica El Carmen, Barranquilla, Colombia; ^8^ Life Science Research Center, Universidad Simon Bolivar, Barranquilla, Colombia

**Keywords:** genomics, neoplasms, research, bibliometrics, Latin America

## Abstract

**Background:** Cancer genomics, as an interdisciplinary research area within the Global Cancer Research agenda, genomics and precision medicine, its important in research and clinical practice in Latin America. To date, there has been no study investigating evolution of this area in this region. The aim of this study was to evaluate for first time, the historical evolution of cancer genomics research in Latin America.

**Methods:** Bibliometric cross-sectional study of documents on cancer genomics published by Latin American authors until 2023 in Scopus was performed. Statistical and visual analysis was performed with R programming language.

**Results:** A total of 1534 documents were obtained. The first document of cancer genomics research was published in 1997, marking the inception of a 26-year evaluation period that extended until 2023. Among the documents, 74.3% (*n* = 1140) constituted original articles, followed by 22.7% (*n* = 349) classified as reviews. International collaboration was observed in 6.5% (*n* = 100) of the articles. Within the compilation of the ten most prolific authors in this region, 90% of them are from Brazil. This observed pattern extends to affiliations as well, wherein the Universidade de São Paulo emerges as the most active institution (*n* = 255 documents). This arrangement firmly establishes Brazil’s prominence as the preeminent country in the region concerning cancer genomics research, showcasing robust collaboration networks both regionally and intercontinentally. An important transition in the studied hot topics over the last 20 years was identified, from the exploration of the human genome and the characterization of genomic and proteomic cancer profiles (1997–2010) to an in-depth investigation of cancer stem cells and personalized medicine (2011–2023). Among the array of cancer types under study, predominant attention has been directed towards breast, lung, prostate, and leukemia.

**Conclusion:** Over the course of the past 26 years, a favorable and notable evolution has characterized cancer genomics research within Latin America, with Brazil leading the way, which possess a robust network of regional and intercontinental collaboration. Furthermore, the lines of research and hot topics have change in harmony with the region’s objectives, strategies, and requisites.

## 1 Introduction

According to the World Health Organization (WHO), cancer ranks among the leading causes of death worldwide, resulting in just over 10 million deaths annually (World Health Organization, 2023). The disease burden resulting from this condition becomes unsustainable for healthcare systems due to the increasing diagnosis of cases at younger ages, which subsequently limits the functional capacity, work activity, and quality of life of the affected individuals (Pan American Health Organization, 2023a). The global health agenda emphasizes the necessity of developing precise and reproducible preventive, diagnostic, and therapeutic tools to aid in reducing the number of new cancer cases and disease burden, particularly in low and middle-income countries (Shah et al., 2019). Approximately one-third of cancer cases worldwide can be prevented, and timely diagnosis and treatment can lead to cures. This field is referred to as Global Cancer Research (National Cancer Institute, 2023b). In Latin America, prostate cancer (15%), breast cancer (14%), colorectal cancer (9%), lung cancer (7%), and stomach cancer (5%) have had the highest incidence in recent years. Therefore, there has been a proposal to place greater emphasis on novel research in these cancers, as they pose the most urgent demand and a significant burden of disease (Shah et al., 2019; Pan American Health Organization, 2023a; World Health Organization, 2023).

Cancer genomics, as an interdisciplinary research area within the Global Cancer Research agenda, genomics and precision medicine, has witnessed significant progress in recent years (National Cancer Institute, 2023a). Through genomics, heritable genetic sequences and biochemical modifications associated with diseases have been identified. Collaborating with areas of work like transcriptomics, proteomics, metabolomics, and others, it has facilitated the identification of therapeutic targets and the successful development of new drugs (Ozdemir et al., 2009; Williams, 2010). However, precision medicine’s limitation lies in the necessity of understanding the heritability pattern of the populations of interest’s ancestry to comprehend their genotypic and phenotypic behavior and devise precise solutions (Naithani et al., 2021). Despite numerous published studies on cancer genomics, the majority originate from high-income countries, resulting in a considerable knowledge gap in low and middle-income countries, such as Latin America, where a diverse ethnic distribution significantly influences cancer’s presentation and prognosis (Wade et al., 2015).

Latin America, a region that has historically faced significant challenges in areas such as funding and research infrastructure, presents a unique genetic diversity. This diversity, a result of the confluence of indigenous, European, and African populations, among others, has a potential impact on susceptibility to cancer and the response to treatments. Understanding and studying this genetic landscape is crucial for the development of a more personalized approach in precision medicine. To date, no study has been conducted that investigates the growth of scientific production, trends, topics of interest, and knowledge gaps in cancer genomics in Latin America. According to the evidence-based research framework (Robinson et al., 2021), it is essential to provide updated data to propose and develop novel research ideas in low-resource setting such as Latin America, especially those that are funded, thus ensuring optimal utilization of time, money, and human resources. Additionally, this integrated information is essential to inform and guide the design of public policy in research, ensuring that the region’s needs and priorities are properly addressed. To solve this knowledge gap, the aim of the study was to carry out a quantitative comprehensive analysis of the historical evolution of cancer genomics research in Latin America up until the year 2023, which allows not only the recognition and appreciation of the advances achieved in the region but also focusing future efforts and resources, and providing novelty evidence to serve as a foundation for the development of future projects aligned with the challenges and goals set by Latin American health.

## 2 Methods

### 2.1 Study design

Bibliometric cross-sectional study.

### 2.2 Source database

A literature search was conducted using Scopus. The Scopus database is the largest database of peer-reviewed literature, which allows access to numerous metrics related to authors, citations, and scientific articles (Romero and Portillo-Salido, 2019). Currently, under the subject area of medicine, it has more than 15,000 indexed journals. For this reason, this database has been previously used for bibliometric analysis (Lozada-Martinez et al., 2023).

### 2.3 Search strategy

A search was designed to identify all scientific articles published, studying cancer from a genomic perspective, involving institutions and authors from Latin America and the Caribbean (identified by their country of affiliation belonging to Latin America). MeSH terms and synonyms were reviewed and utilized for constructing this search. The search designed and used was: TITLE-ABS-KEY(Genomics) OR TITLE-ABS-KEY (“Structural Genomics”) OR TITLE-ABS-KEY (“Functional Genomics”) OR TITLE-ABS-KEY (“Comparative Genomics”) OR TITLE-ABS-KEY (Epigenomics) OR TITLE-ABS-KEY (Glycomics) OR TITLE-ABS-KEY (“HapMap Project”) OR TITLE-ABS-KEY (“Human Genome Project”) OR TITLE-ABS-KEY (“Imaging Genomics”) OR TITLE-ABS-KEY (Nutrigenomics) OR TITLE-ABS-KEY (Proteomics) OR TITLE-ABS-KEY (Proteogenomics) OR TITLE-ABS-KEY (“Radiation Genomics”) AND TITLE-ABS-KEY(Neoplasms) OR TITLE-ABS-KEY(Tumor) OR TITLE-ABS-KEY(Neoplasm) OR TITLE-ABS-KEY(Tumors) OR TITLE-ABS-KEY(Neoplasia) OR TITLE-ABS-KEY(Neoplasias) OR TITLE-ABS-KEY(Cancer) OR TITLE-ABS-KEY(Cancers) OR TITLE-ABS-KEY(“Malignant Neoplasm”) OR TITLE-ABS-KEY(Malignancy) OR TITLE-ABS-KEY(Malignancies) OR TITLE-ABS-KEY(“Malignant Neoplasms”). To this search, the Latin American countries were added: Antigua and Barbuda, Argentina, Bahamas, Belize, Bolivia, Brazil, Chile, Costa Rica, Cuba, Dominican Republic, Ecuador, El Salvador, Grenada, Guatemala, Guyana, Haiti, Honduras, Jamaica, Mexico, Nicaragua, Panama, Paraguay, Peru, Saint Lucia, Suriname, Trinidad and Tobago, Uruguay, Venezuela.

### 2.4 Standardization and data collection

Considering that Spanish and Portuguese rank as the most widely spoken languages in Latin America, while English serves as the global language for scientific communication, the search and identification of articles were restricted to these three linguistic mediums. The database yielded results that encompassed various data points, including the year of publication, article title, journal details, article type, keywords, affiliations, author information, citations, scientific collaborations, language preferences, and publication editorial details. This comprehensive search was carried out until 23 June 2023, with a focus on the filters labeled “Humans” and “Journals.” Subsequently, a manual review was performed by two authors where duplicates and those articles that did not belong to the research scope (cancer and genomics) were eliminated, based on their title, abstract, and keywords in Microsoft Office Excel 2016.

Three authors manually reviewed the articles ultimately chosen to carry out data refining and standardization. The article typology involved grouping them into the “article” category, which encompassed all original studies with observational or experimental designs. The “review” category included narrative, systematic reviews, and meta-analyses. On the other hand, the “editorial” category comprised all articles published under that specific typology, while the “letter” category encompassed any other typologies distinct from the previous ones, such as comments, correspondences, letters to the editor, etc. Moreover, affiliations underwent adjustment through the review and corroboration of the reported Latin American affiliations to avoid the inclusion of affiliations from other regions in the analysis.

### 2.5 Evaluated indicators and metrics

To assess the impact of scientific production on cancer genomics by Latin American authors, affiliations, and countries, employment was made of the h-index, m-index, and g-index, along with the aggregate count of citations garnered, where precise data was available for calculation. The h-index is a quantitative bibliometric index that measures the impact of scientific output based on the number of citations received by published articles (Todeschini and Baccini, 2016; Rousseau et al., 2018). For instance, an author has an h-index if h of their *p* papers have received at least h citations. Mathematically, it is expressed as follows:

$$h=\max_{i}\left\{\text{Ci}\geq i\right\}i=1,2,\ldots ,p\wedge C_{i}\geq C_{i}+1$$

Where p = quantity of documents published by the author, and C_i_ denotes the number of citations for the i-th documents, organized in descending sequence. Consequently, an h-index of 10 for an author necessitates the presence of no less than 10 articles, each gathering a minimum of 10 citations.

The m-index (also known as Hirsch’s m-quotient) is a quantitative index that assesses the linear correlation of an investigator’s impact over time (Todeschini and Baccini, 2016; Rousseau et al., 2018). It is calculated using the following equation:

$$m\equiv h^{N}=h\;/\;Y_{\text{aa}}$$

Here, h denotes the h-index of the author, while Y_aa_ represents the author’s academic age, determined by the subtraction of the present year from the year of the author’s first publication (Todeschini and Baccini, 2016; Rousseau et al., 2018). Hence, an author holding an h-index of 10, and aspiring to compute their m-index in 2023 with their inaugural publication dating back to 2018, would yield an m-index of 2 [10/5 years (2023-2018)].

Lastly, the g-index stands as an additional quantitative measure that emerges from the distribution of accumulated citations garnered by an author (g-value), structured so that, when positioned in descending order, they correspond to the g^2^ ranking (Todeschini and Baccini, 2016; Rousseau et al., 2018). For an author to possess a g-index of 6, it is requisite to have accumulated at least 36 citations (g^2^) from their 6 most cited articles (g), without the necessity for all of them to possess a minimum of 6 citations each.

### 2.6 Data analysis and visualization

All publications that satisfied the inclusion criteria, were exported. The bibliometrix package in R was used to calculate quantitative bibliometric indicators (Version 4.3.1) (Aria and Cuccurullo, 2017). Also, this package was used for visual analysis. Various names, misspellings, or variants (e.g., plurals, synonyms) of words may exist in a thesaurus.txt file. Then, manual standardization was carried out to integrate authors, institutions, and keywords. Microsoft Office Excel 2016 was used to analyze the variables collected on the characteristics of studies and authors, calculating the frequency and percentage.

Characterization of scientific production, evaluation of annual scientific growth, assessment of average citations per year, publication frequency and impact indicators adjusted by journals, was conducted. It was identified the most prolific authors, and Lotka’s law was employed to visualize the distribution of publications among authors, along with the production from Latin American affiliations and countries. Furthermore, we described studies with the greatest impact on cancer genomics and visualized the most studied topics in the region. Only documents were included in the analysis where it was possible to identify and corroborate the contribution of a Latin affiliation or author. Therefore, massive collaborations where affiliations are not clearly defined were excluded. Lastly, collaboration networks among countries and authors were constructed, and the relationship between different variables was explored through the three-field plot.

For analysis of research output based on category, the search query was run with specific keywords for each category. For instance, to visualize the most studied topics, synonyms and general terms were edited and removed. These terms included “man,” “woman,” “adult,” “child,” “study,” etc.

### 2.7 Ethical statement

Ethical approval was not required for this study as it did not involve human subjects or animals, and the Scopus database is open-access.

## 3 Results

**FIGURE 1:**
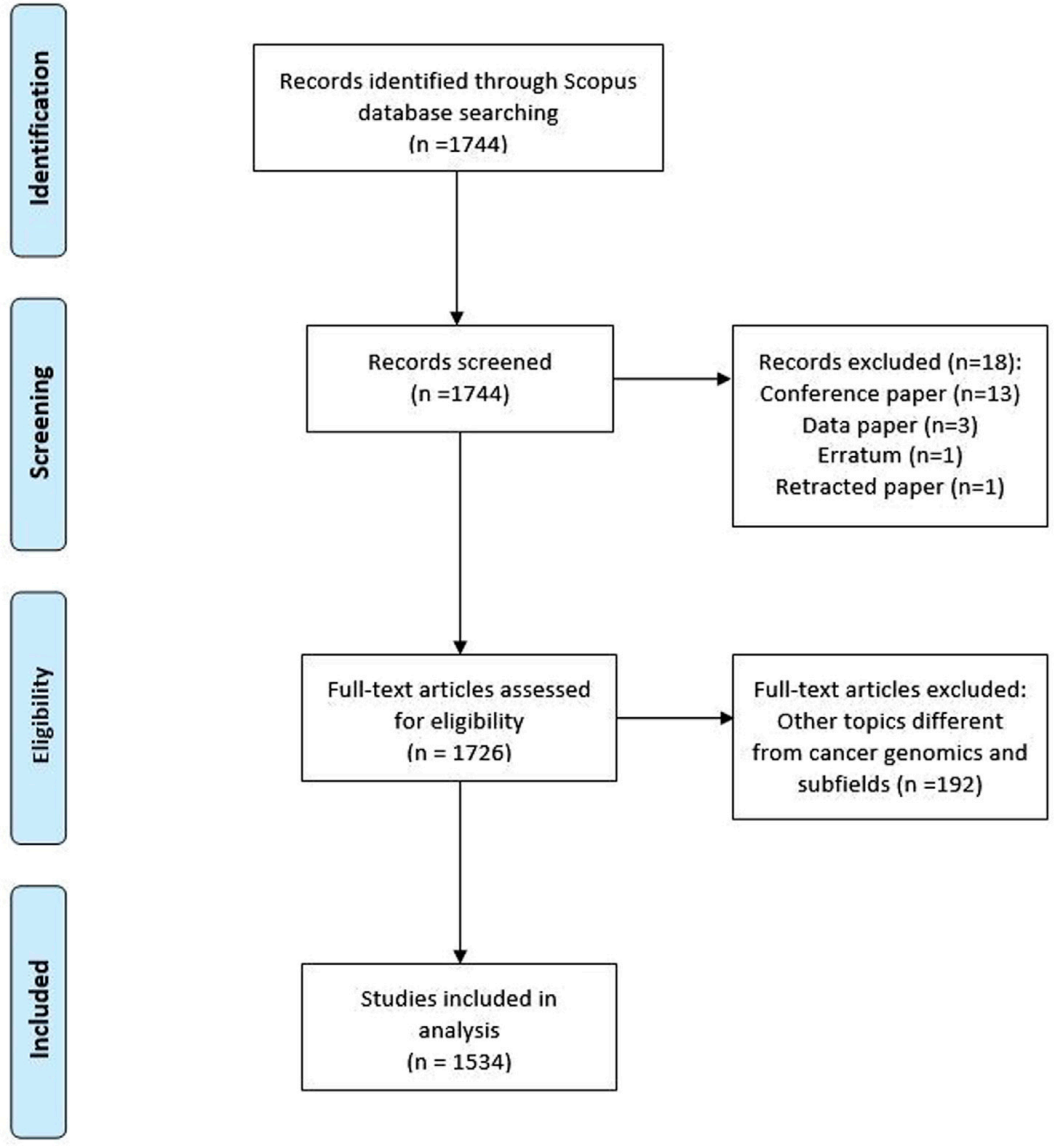
Flowchart of included studies.

Initially, 1744 documents were found. However, after manual review by the authors and verification of the application of inclusion criteria, a total of 1534 documents were obtained (Figure 1). These documents underwent comprehensive analysis involving all relevant variables. The first instance of cancer genomics research being published was identified in 1997, marking the inception of a 26-year evaluation period that extended until 2023. Among the documents, 74.3% (*n* = 1140) constituted original articles, with an additional 22.7% (*n* = 349) classified as reviews. The analysis further revealed a total of 18,225 instances of authorship, yielding an average of 12 authors per article. Notably, international collaboration was observed in merely 6.5% (*n* = 100) of the articles. The growth rate observed on an annual basis stood at 17.35%. The articles exhibited an average age of 6 years, and each article garnered an average of approximately 45 citations (Table 1).

**TABLE 1:** General characteristics of Latin American scientific production on cancer genomics (*N* = 1534).

	n	%
**Document Types**		
Original articles	1140	74.3
Reviews	349	22.7
Editorials	25	1.70
Letters	20	1.30
**Authors**		
Authorships	18,225	-
Authors of single-authored docs (N = 18,225)	36	0.19
**Author collaboration**		
Single-authored documents	42	2.7
Co-Authors per document	12	-
International co-authorships	100	6.5
**Document contents**		
Keywords	9127	-
**Journals**	712	-
**Annual growth rate**	-	17.3
**Document average age (years)**	6	-
**Average citations per document**	45	-

### 3.1 Annual growth

**FIGURE 2:**
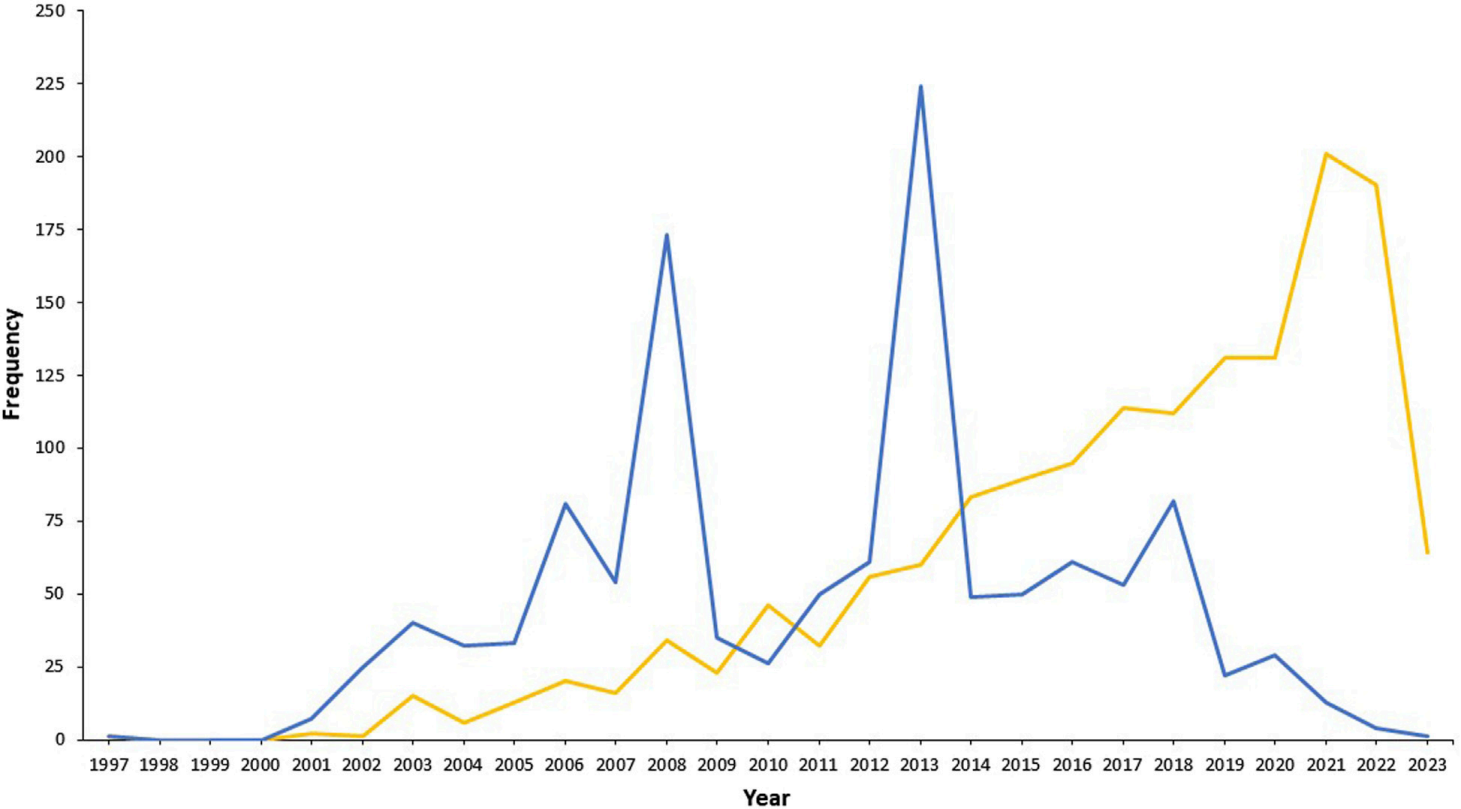
Annual scientific growth of cancer genomics research in Latin America. Frequency of publications per year (Yellow) and average yearly received citations per article (Blue).

Based on the analyzed data, only 4 studies on cancer genomics were published between 1997 and 2002, rendering it the time interval with the lowest production volume. In contrast, the years spanning 2019 to 2022 saw the emergence of the highest volume of documents on this subject (*n* = 652). Notably, 2021 stood out as the most prolific year (*n* = 200), followed by 2022 (*n* = 190) (Figure 2). Of significant importance, a fluctuating pattern emerged in the total number of citations received per year, indicating a tendency towards reduction. This pattern transitioned from 10.8 in 2008 and 20.41 in 2013 to 4.43 and 7.32 in 2019 and 2020, respectively (Figure 2).

### 3.2 Journals

**FIGURE 3:**
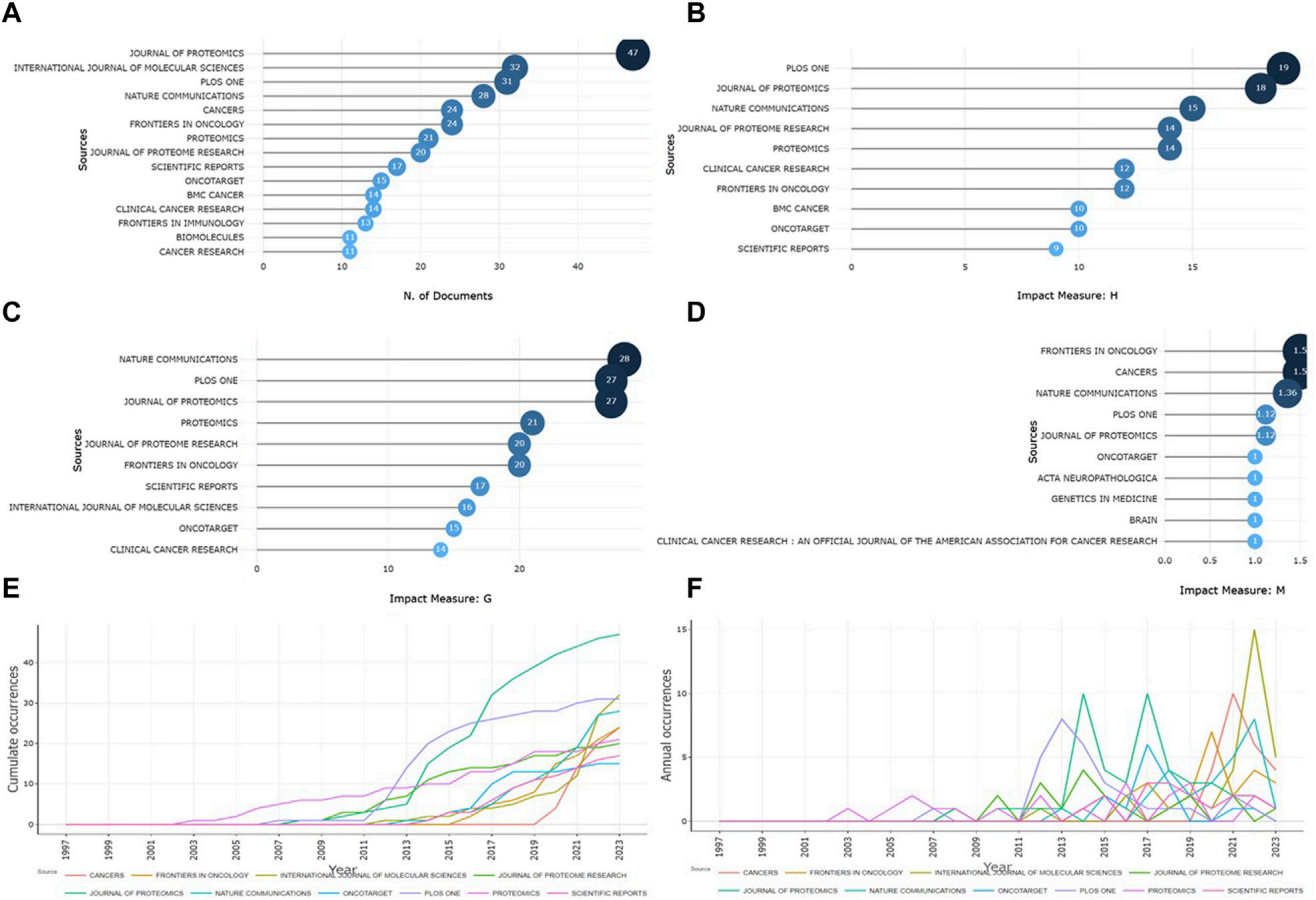
Evolution of journals and impact of their articles on cancer genomics in Latin America. **(A)** Frequency of published articles. **(B)** h-index derived from the articles. **(C)** g-index derived from the articles. **(D)** m-index derived from the articles. **(E)** Cumulative frequency of published articles over time. **(F)** Yearly frequency of articles published on research in cancer genomics in Latin America.

The Journal of Proteomics holds the record for having published the largest number of Latin American articles on cancer genomics to date (*n* = 47; 3.06%). Following closely are the International Journal of Molecular Sciences (*n* = 32; 2.08%) and PLOS ONE (*n* = 31; 2.02%) (Figure 3A). However, upon assessing the impact of these documents on the scientific community, it was found that PLOS ONE exerts the most substantial influence among Latin studies in the field of cancer genomics, as indicated by the h-index (h-index = 19). This is trailed by the Journal of Proteomics (h-index = 18) and Nature Communications (h-index = 15) (Figure 3B). In contrast, when we turn our attention to the g-index and m-index, we observe a different landscape. In terms of the impact derived from their published Latin studies, Nature Communications (g-index = 28) (Figure 3C), Frontiers in Oncology (m-index = 1.5), and Cancers (m-index = 1.5) emerge as the leaders in these metrics (Figure 3D). It is noteworthy that the journals Science (*n* = 7901), Nature (*n* = 5842), and Nature Communications (*n* = 4931) have amassed the highest number of citations from Latin studies on cancer genomics. Upon analyzing the annual evolution of publication volume on this topic since 1997, it was discovered that the International Journal of Molecular Sciences, the Journal of Proteomics, and Cancers exhibit the most robust annual growth (Figure 3E). Among these, the International Journal of Molecular Sciences stands out, achieving the highest volume peak, with publication rates reaching up to 15, 10, and 10 articles per year, respectively (Figure 3F).

### 3.3 Authors

Upon evaluating the five most prolific authors, a notable observation arises, 80% of them hail from Brazil, with the remaining author originating from Mexico. Eliana Saul Furquim Werneck Abdelhay from Instituto Nacional de Cancer, and Fabio Albuquerque Marchi from the Universidade de São Paulo stand as the authors possessing the highest count of documents published in the field of cancer genomics (both having 36 documents). Similar, Fabio Albuquerque Marchi and Eliana Saul Furquim Werneck Abdelhay possess the studies characterized by the highest h-index (h-index of 17 and 16, respectively). In contrast, Sergio Rodríguez-Cuevas (from Instituto de Enfermedades de la Mama - Sociedad Mexicana De Oncología), and Eliana Saul Furquim Werneck Abdelhay claim the highest g-indices (indices of 24 and 22, respectively). The most noteworthy m-index scores were attained by Fabio Albuquerque Marchi (m-index = 1.7) (Table 2).

**TABLE 2:** Description of the most prolific authors and the impact of their studies on cancer genomics in Latin America.

Author	Documents on cancer genomics	h index	g index	m index	Total citations	Affiliation	Country
Eliana Saul Furquim Werneck Abdelhay	36	16	22	1	882	Instituto Nacional de Cancer	Brazil
Fabio Albuquerque Marchi	36	17	16	1.7	724	Universidade de São Paulo	Brazil
Adriana Franco Paes Leme	20	13	14	1	780	Laboratório Nacional de Biociências	Brazil
Sergio Rodríguez-Cuevas	12	10	24	0.5	1537	Instituto de Enfermedades de la Mama - Sociedad Mexicana De Oncología	México
Daniela Campos Granato	8	8	10	0.8	263	Laboratório Nacional de Biociências	Brazil

**FIGURE 4:**
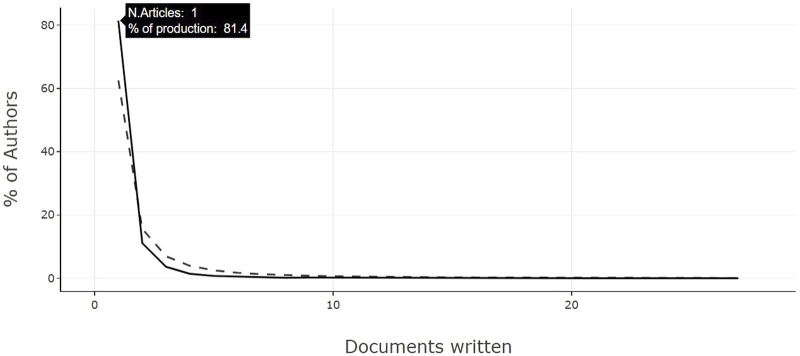
Lotka’s Law depicting the distribution of publications by authors.

Lastly, authors Sergio Rodríguez-Cuevas and Eliana Saul Furquim Werneck Abdelhay garnered the highest citation counts within the sphere of cancer genomics studies (1537 and 882 citations, respectively). Upon the application of Lotka’s law, a distinctly heterogeneous distribution of authors’ scientific output becomes apparent (Figure 4). The findings reveal that a substantial 81.4% (*n* = 14,835) of authors contributed a sole article on this subject. In contrast, a meager less than 2.5% [*n* = 455 (approximately)] ventured into publishing five or more articles, while less than 1% [*n* = 182 (approximately)] pursued the creation of 10 or more articles.

### 3.4 Affiliations

**FIGURE 5:**
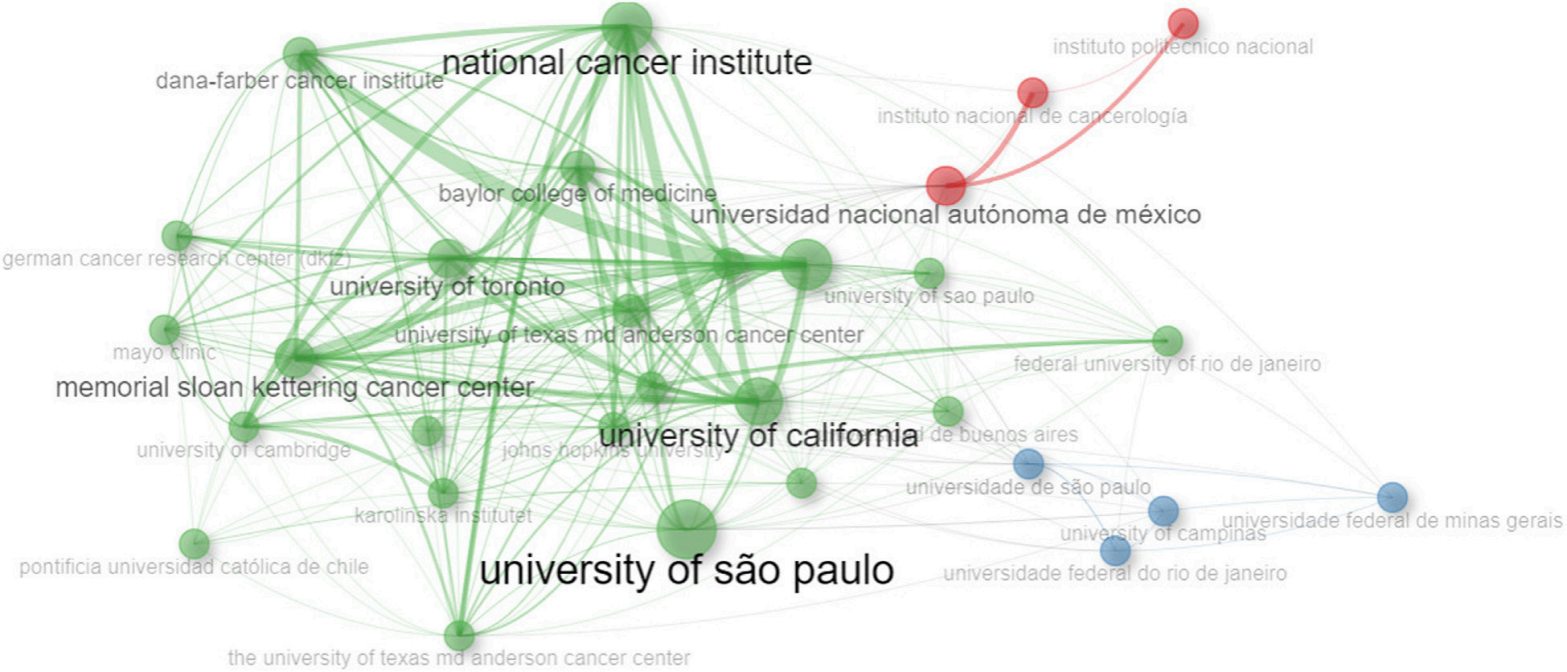
Collaboration networks and strength of regional and intercontinental collaboration on cancer genomics in Latin America, involving Latin American affiliations.

When the scientific output of affiliations is examined, the Universidade de São Paulo emerges as the most prolific entity (*n* = 255; 16.62% of the documents), followed by the Universidad Nacional Autónoma de México (*n* = 109; 7.10% of the documents), and the Universidade Federal do Rio de Janeiro (*n* = 96; 6.25% of the documents). Upon the assessment of the impact of articles published by these institutions, it becomes evident that the Universidade de São Paulo, Universidade Estadual de Campinas, and Universidade Federal do Rio de Janeiro boast the highest h-indices (h-indices of 47, 24, and 23, respectively). Conversely, in the context of the historical evolution of these institutions, an observation is made regarding the modest nature of cancer genomics research until the year 2010 (excluding the Universidade de São Paulo, which had published 35 documents). However, in 2017, there was a significant surge, resulting in the most productive phase between 2017 and 2023 (in all cases), encompassing a range of 20–156 publications (Table 3). Importantly, robust international collaboration was evident between the Universidade de São Paulo and American institutions (such as the National Cancer Institute and the University of California) (Figure 5). Meanwhile, in Mexican institutions, national collaboration held prominence, particularly notable in partnerships between the Universidad Nacional Autónoma de México, Instituto Nacional de Cancerología (Mexico), and Instituto Politécnico Nacional.

**TABLE 3:** Most prolific Latin American affiliations in cancer genomics.

Affiliation	Documents over time				Total documents on cancer genomics	h index	Country
	1997–2003	2004–2010	2011–2017	2018–2023			
Universidade de São Paulo	0	35	64	156	255	47	Brazil
Universidad Nacional Autónoma de México	0	6	22	81	109	22	Mexico
Universidade Federal do Rio de Janeiro	0	16	35	45	96	23	Brazil
Universidade Federal de Minas Gerais	0	3	22	41	66	17	Brazil
Instituto Nacional de Cancerología	0	3	17	22	42	20	Mexico
Universidad de Buenos Aires	0	5	6	26	37	18	Argentina
Instituto Politécnico Nacional	0	2	14	20	36	17	Mexico
Universidad de Chile	2	7	3	23	35	13	Chile
Pontificia Universidad Católica de Chile	0	1	13	20	34	19	Chile
Universidade Estadual de Campinas	0	1	7	25	33	24	Brazil

### 3.5 Countries

When country-specific production is examined (taking individual production into account), it emerges that Brazil (*n* = 903), Mexico (*n* = 360), and Argentina (*n* = 156) are the most active nations in this research field. They also lay claim to the highest impact in cancer genomics research in Latin America (with h-indices of 76, 46, and 35, respectively). Following a pattern akin to the most prolific affiliations, there existed a phase of modest production until the year 2010 (excluding Brazil, Mexico, and Argentina, which had already published over 20 documents). Subsequently, there was a notable surge in production in 2017, followed by a marked peak in 2023 (this trend manifested across all countries). However, some nations displayed notably restrained behavior, like Peru, Uruguay, Cuba, Ecuador, and Costa Rica. These countries published no more than two documents annually until 2017, and by 2023, no more than four annually (Table 4).

**TABLE 4:** Most prolific Latin American countries in cancer genomics.

Country	Documents over time				Total documents on cancer genomics	h index	SCP
	1997–2003	2004–2010	2011–2017	2018–2023			
Brazil	6	81	306	510	903	76	488
Mexico	9	30	138	183	360	46	178
Argentina	2	27	44	83	156	35	67
Chile	5	16	39	94	154	34	40
Colombia	0	3	36	59	98	23	33
Peru	0	1	11	22	34	15	3
Uruguay	2	7	14	7	30	14	5
Cuba	1	5	9	9	24	10	9
Ecuador	0	1	2	10	13	6	7
Costa Rica	0	0	3	7	10	5	3

SCP: single country publication.

*Individual production was counted; therefore, a document might be counted multiple times according to international collaboration.

**FIGURE 6:**
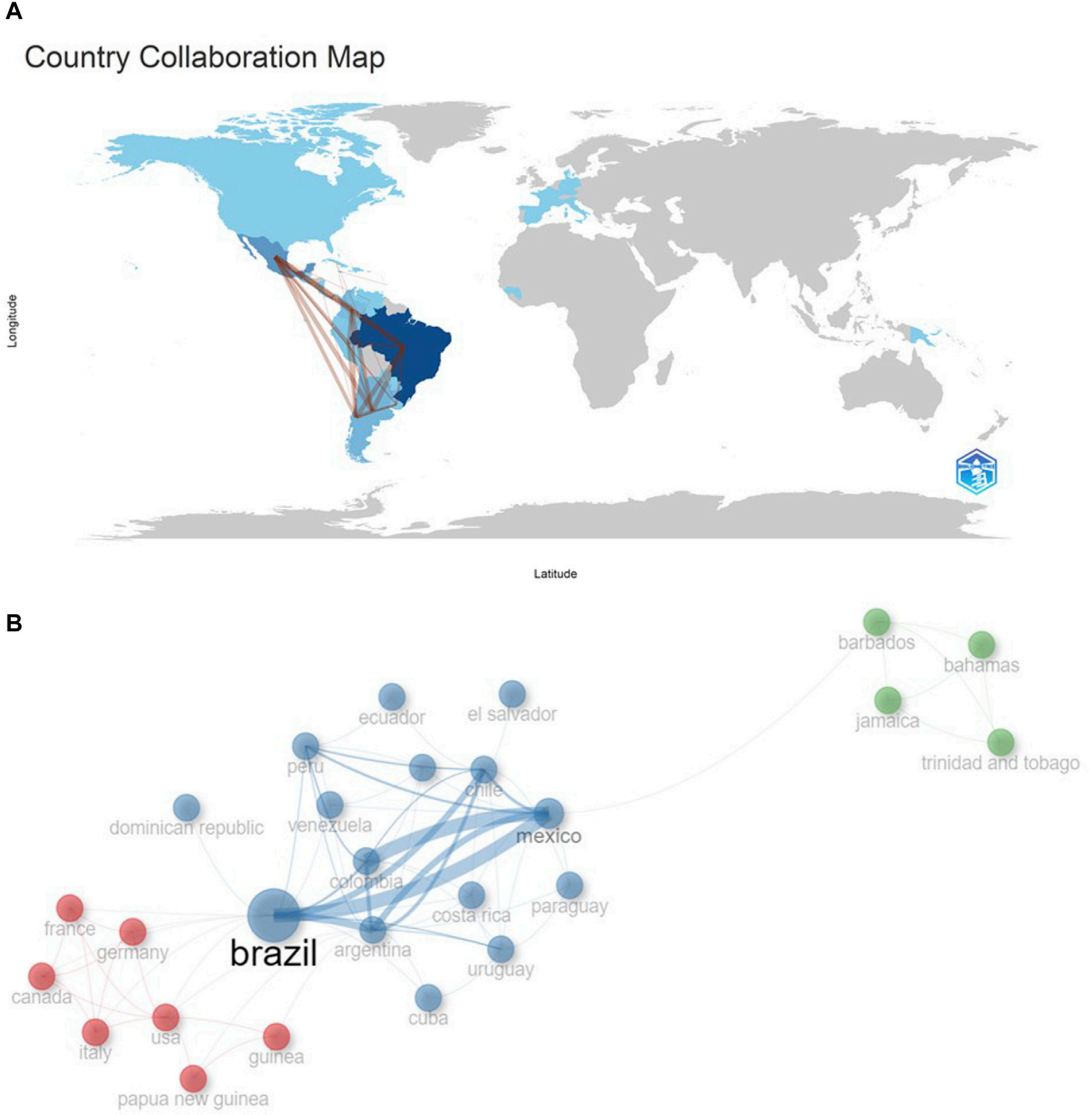
International collaboration networks. **(A)**. Frequency of regional collaboration. **(B)**. Strength of regional and intercontinental collaboration, involving Latin American countries.

As for collaboration and the strength of collaboration among nations, it has been discerned that Brazil holds the largest regional collaboration network, particularly robust with Mexico, Argentina, and Chile (Figure 6A). Mexico, in addition, maintains a formidable partnership with Colombia and Argentina. Peru, in essence, has fortified its collaboration with Chile, Mexico, and Colombia (Figure 6B).

### 3.6 Hot topics and research lines

**FIGURE 7:**
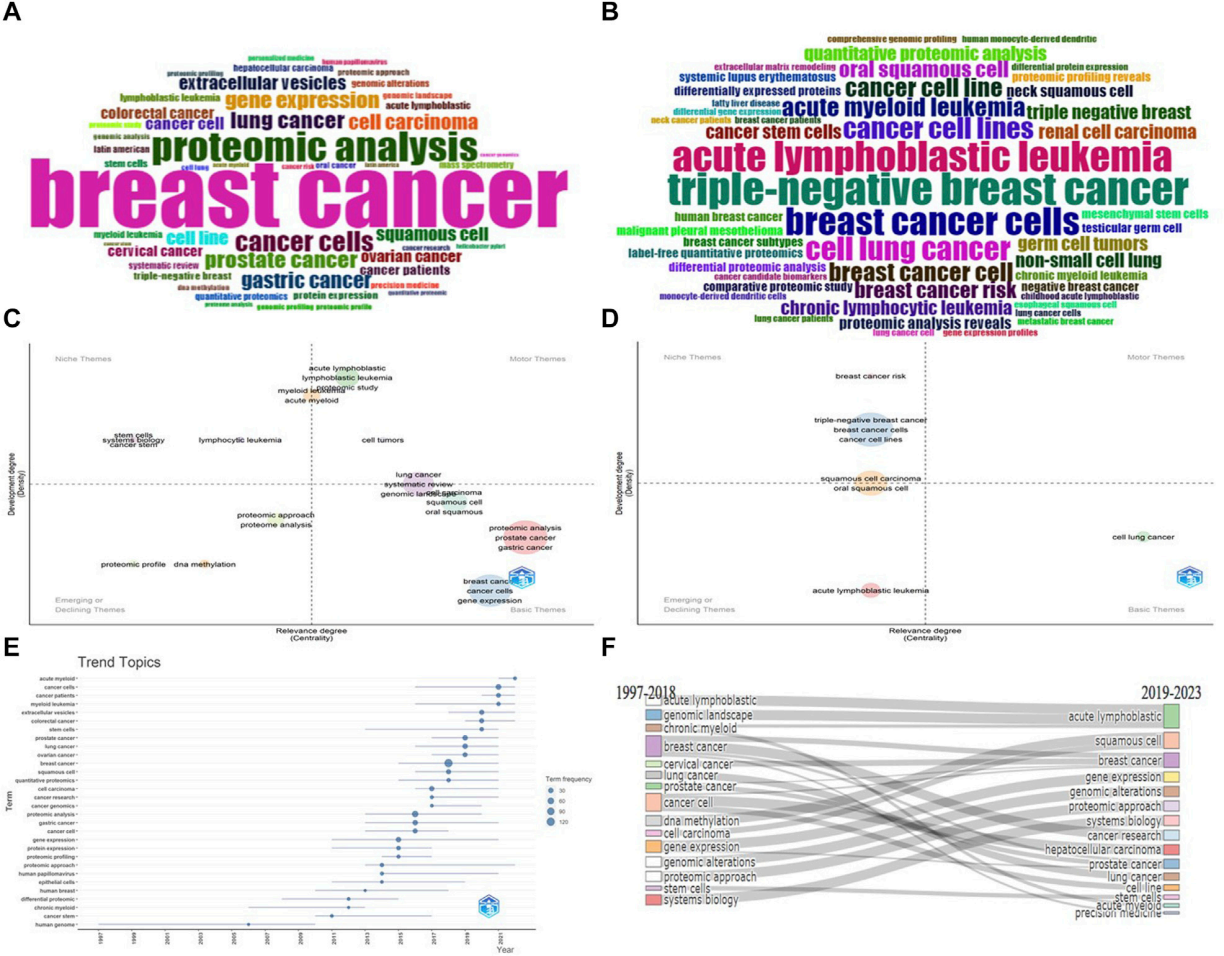
Predominant mixed topics and research lines in cancer genomics in Latin America. **(A)** Wordcloud of most frequent bigrams. **(B)** Wordcloud of most frequent trigrams. **(C)** Thematic map of most relevant bigrams. **(D)** Thematic map of most relevant trigrams. **(E)** Evolution of hot topics over time. **(F)** Thematic transition from 1997 to 2023.

Exploring the region’s most frequently studied hot topics, the grouping of title words into bigrams revealed that breast cancer (*n* = 121), proteogenomic analysis (*n* = 53), and cell line (*n* = 42) took the lead in this domain (Figure 7A). Conversely, grouping by trigrams unveiled squamous cell carcinoma (*n* = 21), triple-negative breast cancer (*n* = 14), and acute lymphoblastic leukemia (*n* = 13) as the most prevalent (Figure 7B). Moreover, when thematic maps were created for unigrams, bigrams (Figure 7C), and trigrams (Figure 7D), the foundational themes in cancer genomic research in Latin America became evident: breast cancer, lung cancer, gastric cancer, oral cancer, and squamous cell carcinoma. Emerging themes encompass DNA methylation, acute and chronic lymphoblastic leukemia, and the identification of proteomic or proteogenomic profiles. When assessing the evolution of the most active topics over time (identified by the most frequently used keywords), a trend emerged wherein breast cancer, while remaining the most researched subject, saw a growing surge in research on colorectal cancer, lung cancer, and cancer stem cells in recent years (Figure 7E).

An analysis of research pathways developed through collaboration, both at the level of countries and affiliations, showed that while Brazil demonstrates a strong inclination towards cancer proteogenomic analysis, countries such as Mexico, Argentina, and Colombia focus on gene expression and biomarker identification in cancer. Similarly, emerging countries in cancer genomic research, including Colombia, Peru, and Uruguay, enhance their research trajectories by studying cancer cells. Lastly, a diverse range of research directions was identified among the most prolific affiliations, with significant emphasis on the utilization of cancer cell lines, human studies, biomarker identification, and the molecular and proteomic expression of cancer. This transition in research directions reflects a shift from investigating the human genome, gene expression, and genomic alterations between 1997 and 2018, towards more precisely defined research paths concentrating on cancer types and localization, as well as the application of cancer stem cells and precision medicine from 2019 onward (Figure 7F).

### 3.7 Most relevant studies

Finally, upon examination of the most frequently cited studies in cancer genomics involving Latin American authors, affiliations, or countries, Brazil’s participation was evident in 70% of these articles, while Mexico contributed to the remaining 30%. Interestingly, none of these articles involved collaboration between more than one Latin American country. All these documents were published in journals that, as of the year 2023 (based on 2022 metrics from Scimago Journal & Country Rank), hold Q1 status, with h-indices reaching up to 1331, exemplified by journals like Nature, Science, and Cell. Fifty percent of these documents were published between 2008 and 2013, with the other half spanning from 2016 to 2020. The most influential document garnered a maximum of 4636 citations. The Universidade de São Paulo’s involvement constituted 60% of these documents, whereas Mexico’s contribution displayed diversity, featuring single authorships per institution from the country. Topics addressed in these documents encompass integrated genomic analyses, gene expression profile descriptions, and molecular and genomic characterizations of diverse cancer types (esophagus, breast, and cervix) (Table 5).

**TABLE 5:** Most cited articles on cancer genomics, involving authors, affiliations, and Latin American countries.

Article name (year of publication)	Journal characteristics				Latin American participating countries	Latin American participating affiliations	Citations
	Name	SJR^a^	h index^a^	Quartile^a^			
An Integrated Genomic Analysis of Human Glioblastoma Multiforme (2008)	Science	13.32	1283	Q1	Brazil	Universidade de São Paulo	4636
The Cancer Genome Atlas Pan-Cancer analysis Project (2013)	Nature Genetics	16.73	621	Q1	Brazil	Universidade de São Paulo	4409
Inferring tumour purity and stromal and immune cell admixture from expression data (2013)	Nature Communications	5.11	466	Q1	Mexico	Tecnologico de Monterrey	3935
The Somatic Genomic Landscape of Glioblastoma (2013)	Cell	26.49	856	Q1	Brazil	Universidade de São Paulo	3190
The Immune Landscape of Cancer (2018)	Immunity	14.79	436	Q1	Brazil	Universidade de São Paulo	2502
TCGAbiolinks: an R/Bioconductor package for integrative analysis of TCGA data (2016)	Nucleic Acids Research	8.23	607	Q1	Brazil	Universidade de São Paulo	1578
Pan-cancer analysis of whole genomes (2020)	Nature	20.95	1331	Q1	Mexico	Instituto Carlos Slim de la Salud	1183
Integrated genomic characterization of oesophageal carcinoma (2017)	Nature	20.95	1331	Q1	Brazil	Barretos Cancer Hospital/Universidade de São Paulo	1071
Sequence analysis of mutations and translocations across breast cancer subtypes (2012)	Nature	20.95	1331	Q1	Mexico	Instituto Nacional de Medicina Genómica/Instituto de Enfermedades de la Mama FUCAM/	953
Integrated genomic and molecular characterization of cervical cancer (2017)	Nature	20.95	1331	Q1	Brazil	Barretos Cancer Hospital/Universidade de São Paulo	871

SJR, scimago journal ranking.

^a^ Metrics based on SJR 2022.

## 4 Discussion

Bibliometrics is a mixed quantitative and qualitative method, although essentially quantitative, that employs metrics and mathematical calculations to identify patterns and trends in academia based on citations and variables related to scientific publication and dissemination (Donthu et al., 2021). In this way, it is possible to determine and visualize the impact, evolution, correlation, occurrence, and co-occurrence of massive data in research and scientific publication (Donthu et al., 2021). Through this methodology, it is possible to assess what, how, who, and to what extent research has been conducted in a specific area of knowledge. In practical terms, it allows for understanding knowledge gaps, pluralism, common scientific techniques and procedures, achievement of research objectives, and the proposal of future directions (Todeschini and Baccini, 2016).

Through this method, the landscape and evolution of over 20 years of cancer genomics research in Latin America were revealed by this study for the first time. The relevance and significance of this data lie in the evaluation of the pertinence, rigor, and impact of Latin American science, which aims to progress in the study of cancer that present a substantial burden in the region (National Cancer Institute, 2023b; Pan American Health Organization, 2023a; National Cancer Institute, 2023a). The capacity for execution, collaboration, and influence of the scientific evidence developed by Latin authors, who encounter significant limitations in terms of infrastructure and funding, can be observed as well (Pan American Health Organization, 2023c). Additionally, and of even greater importance, answers to specific questions posed by Global Cancer Research are provided by this data. For instance, the advancement in the addressing of cancer and precision medicine as a health priority in low- and middle-income countries, such as those in Latin America, can be highlighted (Alvarez-Gomez et al., 2021; National Cancer Institute, 2023b; National Cancer Institute, 2023a). Furthermore, the correlation of whether the focus and interests of cancer researchers in the region align with the needs of the population—such as the most common, prevalent, costly, and deadly cancers—can be determined (Pan American Health Organization, 2023b). Moreover, support is offered for the development of an evidence-based control plan, the provision of open access data and records, the establishment of biobanks for future research, and the study of communities at higher risk of cancer to facilitate the development of diagnostic techniques and personalized therapies (National Cancer Institute, 2023b; National Cancer Institute, 2023a; Pan American Health Organization, 2023b).

Overall, significant progress in terms of publication volume, particularly over the last 5 years, has been identified within the realm of scientific production concerning this topic. This progress primarily comprises original studies, featuring limited international collaboration, yet exhibiting a noteworthy average author count per article (*n* = 12). Moreover, it was observed that the majority of these documents were published in high-impact journals. These journals have wielded influence within the scientific community and garnered a minimum of 20,000 citations. The region’s most influential authors hail from Brazil. According to the metrics under evaluation, their documents have borne significant impact. However, in alignment with the outcomes of Lotka’s law, more than 80% of the authors contributing to the total corpus of documents published within the region have only published once. This phenomenon suggests a fragmentation in the continuity of cancer research trajectories. Of the most prolific authors within the region, 80% are of Brazilian origin. This is despite the fact that the most prolific affiliations are situated in Brazil, Mexico, Chile, and Argentina. Nevertheless, Brazil lays claim to a considerably greater number of documents (*n* = 903) in comparison to other countries. It also boasts a more substantial impact of its documents (h-index = 76). This pattern and trajectory of scientific growth have positioned Brazil as the most actively engaged country in cancer genomics research in Latin America. Lastly, despite the occurrence of a transition in focal themes and research directions, attention has been directed toward the depiction of genomic profiles of diverse cancer types with regional relevance. This is in addition to the emergent interest in the utilization of cancer stem cells. Particularly, an active and frequent combination of research methods was identified in the study of cancer in Latin America, involving genomics and other omics. One of the most common approaches is the mixed use of proteomics alongside genomics (proteogenomics), as illustrated in Figure 7.

The predominance of original studies in this field indicates the interest of Latin authors in the generation of novel knowledge in cancer, which has been experiencing gradual growth. Notably, there exists a production peak commencing from the year 2020, aligning with the timeframe of the Coronavirus Disease 2019 (COVID-19) pandemic. This may have facilitated both national and international investment towards the enhancement of infrastructure (Pan American Health Organization, 2023c), yielding a direct impact on the progression of cancer research. This serves as a demonstration that this form of funding does indeed wield a direct influence on the advancement of biomedical research in Latin America (Torres et al., 2017). In due course, these pioneering studies epitomize tangible progress. This could elucidate the reason behind the successful publication of these documents in globally esteemed and rigorous journals like Nature or Science. This mechanism underscores how the scientific community has acknowledged the frequency and impact of these documents, as evidenced by citations received and evaluated metrics. Regrettably, fewer than 20% of the identified authors have contributed more than one document on this subject. Probably, this implies a transient or fragmented inclination towards cancer research by young researchers. This scenario might find explanation in factors such as the time imperative for long-term investment in certain instances, the substantial limitations in acquiring funding, and the scarcity of highly skilled mentors and robust labs to execute profoundly intricate techniques (Seruga et al., 2014).

Notwithstanding the aforementioned, there exist pathways capable of aiding in the surmounting of these barriers. An illustration of this can be found in the case of the Universidade de São Paulo, which exhibited robust regional and intercontinental collaboration, particularly with high-income countries. This, eases the formulation and execution of studies characterized by the utmost quality (Henshaw, 2013; Åstedt-Kurki and Kaunonen, 2019). Likewise, through the reinforcement of such collaborations, Mexico has managed to attain a significant number of documents in recent years. Consequently, the relevance and influence of Latin American countries experience a proportional rise in accordance with the research advancements of their respective institutions. It is for this reason that Brazil and Mexico, possessing the most actively engaged affiliations in cancer genomics research, emerge as the most distinguished nations within the region concerning this subject.

On the other hand, albeit somewhat more intricate for discussion and explication, the hot topics and lines of research developed within the region are encountered. Key statistics and the most pertinent types of cancer in the region have been presented by the Pan American Health Organization (PAHO): 1) Commoner among men and women (breast, lung, colorectal, and prostate); 2) More lethal among men and women (lung, breast, prostate, and colorectal); and 3) Preventable (cervix) (Pan American Health Organization, 2023b). Undoubtedly, the curiosity and general interest in discerning potential solutions in other cancer types, which could be extrapolated to the most momentous cancers in the region, constitute an acceptable stance. However, a significant emphasis on investigating and proffering strategies, plans, and solutions for the most relevant neoplasms in Latin America must be placed (National Cancer Institute, 2023b; Pan American Health Organization, 2023b). This study ascertained the existence of active lines of research concerning the most pertinent cancers in the region, culminating in a noteworthy contribution to scientific evidence. Prevalent in the region’s research pursuits, breast cancer takes precedence, closely followed by leukemia (despite its absence among the most prevalent and lethal cancers). Encouragingly, emerging niche themes, indicative of advancements in the utilization of more intricate cellular and molecular methodologies, such as the employment of cancer stem cells and epigenetic analysis of cancer, have been discerned. The transition in research trends from 2 decades past to the present has evolved in accordance with the region’s requisites. Notably captivating to myriad scholars in the current epoch is the concept of precision medicine. It is imperative to accentuate that these topics of significant interest are progressively appealing to emerging countries (such as Colombia and Peru) (Tekola-Ayele and Rotimi, 2015), nations that aspire to wield influence over research endeavors within the region and around the world.

Conclusively, the most compelling argument in favor of executing collaborative studies with high-income countries lies in the evidence of publishing documents of the utmost caliber within the world’s most impactful journals (Henshaw, 2013; Åstedt-Kurki and Kaunonen, 2019). Robust and extensive regional and intercontinental collaboration facilitates the addressing of barriers that may arise during research, encompassing limited sample size, inadequate funding for procuring essential resources, access to high-level measurement instruments and costly molecular techniques, as well as elevated interdisciplinary academic and scientific discourse, among others (Séguin et al., 2008; Mitropoulos et al., 2015; Ioannidis, 2018). In this study, it was noted that Brazil and Mexico have emerged as the most actively engaged nations in this form of collaboration. Analogously, they have asserted their influence as the most impactful countries, given their possession of the highest number of documents and the most substantial research impact in the field of cancer genomics in Latin America. As elucidated, these documents successfully secure publication in such journals due to their establishment of steadfast collaborations with researchers of the utmost caliber. This, in turn, substantially enhances methodologies and research practices, thereby yielding evidence that is more transparent, valid, and reproducible (Ioannidis, 2018). This trajectory should motivate other nations to reinforce their networks of international collaboration with the aim of augmenting the quality of their research endeavors.

Although there are bibliometric studies that have evaluated patterns and trends in cancer-specific research, the evolution of cancer genomics research has never previously been studied. Then, our analysis provides comprehensive insights into the historical evolution of cancer genomics research in Latin America over a span of more than 20 years have been successfully provided by this study for the first time. Additionally, it entailed the characterization of authors, affiliations, countries, prevailing topics of interest, research trends, and the most impactful documents within the region concerning this subject. Moreover, it shed light on the journals that hold significant interest for these authors, alongside the potential impact exerted by these studies. Consequently, a lucid demonstration was made regarding the outcomes of years of dedication by diverse authors and the dedication of institutions and nations towards the advancement and support of cancer genomics research. These efforts are in alignment with the challenges and necessities articulated by regional and international scientific societies. But also, these data serve as a basis for the approach of new guidelines, strategies and policies in science, allowing to compare funding, progress and research, with the priorities in cancer genomics and precision medicine in this region.

The low international collaboration rate, standing at 6.5%, represents an unexploited opportunity to enrich research in the field of cancer genomics. The incorporation of diverse international perspectives and resources could bring greater depth and variety to the studies. Encouraging international collaboration could not only improve the quality of the research but also expand its reach and resonance in the global scientific community.

On the other hand, the heterogeneous distribution of scientific production, where a notable 81.4% of authors have contributed with a single article, signals a possible lack of continuity or depth in individual research. This trend could limit the development of solid and coherent lines of research. The promotion of closer collaboration and continuous research among authors could mitigate this problem, allowing for greater cohesion and strength in the studies, and fostering a more significant and sustained contribution in the field of cancer genomics.

As limitations of this study, one must note that utilizing data deposited in the Scopus database as the unit of analysis links the quality directly to the accuracy of the records. Consequently, an absolute precision of the scientific production in cancer genomics in Latin America is not mirrored by these results. Similarly, biases intrinsic to scientific publication exist, and these cannot be modified by the researchers executing this study.

## 5 Conclusion

Over the course of the past 26 years, a favorable and notable evolution has characterized cancer genomics research within Latin America, with Brazil and Mexico leading the way, which possess a robust network of regional and intercontinental collaboration. Furthermore, the lines of research and hot topics have unfolded in harmony with the region’s objectives, strategies, and requisites. Consequently, an even more pronounced positive influence is envisaged in the forthcoming years regarding the exploration of cancer genomics in the context of Latin America. The combination of positive evolution in cancer genomics research in Latin America, with a focus on genetic diversity and the identified areas for improvement, could position the region as a leader in this vital field. Continuous attention to genetic diversity will not only enrich scientific research but also contribute to developing more effective and personalized treatments for cancer, leveraging the unique potential of the region and contributing significantly to global science.

## Data Availability

Publicly available datasets were analyzed in this study. This data can be found here: Scopus.
